# An on-chip instrument for white blood cells classification based on a lens-less shadow imaging technique

**DOI:** 10.1371/journal.pone.0174580

**Published:** 2017-03-28

**Authors:** Yuan Fang, Ningmei Yu, Runlong Wang, Dong Su

**Affiliations:** 1 School of Automation and Information Engineering, Xi’an University and Technology, Xi’an, Shaanxi Province, China; 2 School of Electrical and Electronic Engineering, Baoji University of Arts and Sciences, Baoji, Shaanxi Province, China; Universita degli Studi di Milano-Bicocca, ITALY

## Abstract

Routine blood tests provide important basic information for disease diagnoses. The proportions of three subtypes of white blood cells (WBCs), which are neutrophils, monocytes, lymphocytes, is key information for disease diagnosis. However, current instruments for routine blood tests, such as blood cell analyzers, flow cytometers, and optical microscopes, are cumbersome, time consuming and expensive. To make a smaller, automatic low-cost blood cell analyzer, much research has focused on a technique called lens-less shadow imaging, which can obtain microscopic images of cells in a lens-less system. Nevertheless, the efficiency of this imaging system is not satisfactory because of two problems: low resolution and imaging diffraction phenomena. In this paper, a novel method of classifying cells with the shadow imaging technique was proposed. It could be used for the classification of the three subtypes of WBCs, and the correlation of the results of classification between the proposed system and the reference system (BC-5180, Mindray) was 0.93. However, the instrument was only 10 × 10 × 10 cm, and the cost was less than $100. Depending on the lens-free shadow imaging technology, the main hardware could be integrated on a chip scale and could be called an on-chip instrument.

## Introduction

In medical fields, classification of white blood cells (WBCs) is a basic and important method to diagnose diseases [1–4]. There are three major types of WBCs (neutrophils, monocytes, lymphocytes) in human whole blood, and the proportions of these different types is basic information for medical diagnosis [5–7]. To determine the proportions of the different types of blood cells, classification is performed by microscopy, electrical impedance or laser light scattering. The traditional method uses a blood smear and Wright's staining, and the different type of white cells are counted under the objective lens of a microscope [8]. In this method, microscopic images of stained cells are classified, and the staining and counting are time consuming. Recently, flow cytometry and hematology analyzers have been used instead of the manual method. These instruments use flowing liquid to focus on the cells that go through a laser beam and then capture spectral information, such as the forward and side scattering [9], or they drive cells through the micro-orifices to obtain the impedance change, which is called the Coulter effect [10]. The light scattering and implement changes provide information about the cells, such as size, internal complexity and granularity of cells, which enables the sorting of different cell types.

At present, point-of-care testing (POCT) is a trend in medical detection for early detection and early disease treatment, but it could also be used in underdeveloped areas or outside the laboratory for medical testing [11], for example, in battlefields and earthquake or natural disaster sites. There are imperfect health care systems in these areas, but a large number of diseases must be diagnosed. Recently, lab-on-a-chip techniques have made it possible to provide miniature low-cost instruments for blood analysis. There were several reports that utilize complementary metal oxide semiconductor (CMOS) image sensors and microfluidics to observe cells [12, 13].

Mohendra Roy [14] found that cells of different sizes demonstrated different diffraction patterns, and that characteristic was utilized to calculate the size of each cell. Based on the principles of biology, T. Shibata and his team proposed a technique based on edge feature images and direction as a key in video for extraction and representation. The projection of principal-edge distribution (PPED) was a better representative method of global features, which was proposed by M. Dr. Yagi [15]. It could represent a 64×64 image as a 64-dimensional vector. Analysis using PPED features, image classification and recognition tasks, such as face detection and recognition, was more complex when better results were obtained.

The size of white blood cells was an important basis characteristic for classifying WBCs, it was widely used in the automatic hematology analyzers. To prove that the diffraction patterns of different size of objects were different, Mohendra Roy [14] used two types of micro beads (9.7 μm and 21 μm beads) to make an experiment. The results showed that the diffraction fringes of different sizes of micro beads were different, and the conclusion was used to classify the three subtypes of WBCs. However, the method proposed by Mohendra Roy cannot distinguish different types of cells in the same size. In this paper, we proposed a novel method to classify the WBCs. The method utilized the pattern of WBCs to detect the comparability. In order to reduce the effect caused by diffraction and incoherent source, the blood smear was mounted close to the surface of the image sensor [13]. Then, the images of the WBCs were segmented from the whole blood smear shadow images captured by the image sensor, and they were reshaped to 64 × 64 images to extract the PPED feature vector. To verify the effectiveness of the PPED algorithm in the system, we carried out experiments. First, blood smear images captured by the CMOS shadow imaging system were compared with the images captured by the microscope. Next, the changes in the PPED characteristic vector of the same cells under 40×, 10× and 4× objective lenses were studied. There were no obvious differences in the PPED feature vectors at different magnifications, and the cell images captured by the CMOS shadow imaging system and the images captured by 4× objective lens were similar. Finally, the blood smear images were used for WBCs classification, and the result was compared with that of a hematology analyzer (BC-5180, Mindray, China).

## Materials and methods

### Instrument setup

**Fig 1:**
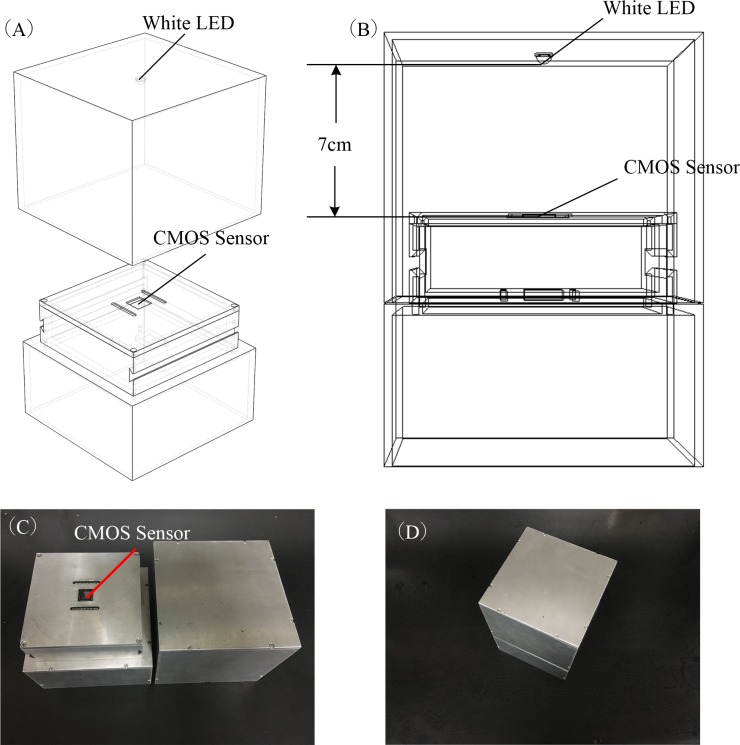
CMOS shadow imaging instrument. (A) Diagram of the CMOS shadow imaging instrument. (B) Diagram of the instrument when working. (C) Photo of the CMOS shadow imaging instrument. (D) Photo of the system when working.

An algorithm was used in our shadow imaging system, and the result of WBC classification was compared with the results of the automated hematology analyzer. Here, a novel instrument (Fig 1) that we invented is used to obtain the shadow imaging of the blood smear. The instrument contains three parts: a CMOS sensor, white light emitting diode (LED) illumination and image processing software. The CMOS sensor was an Aptina MT9J003 (Micron Technology, USA), the pixel size was 1.67 μm, and the active pixel was 3856 H × 2764 V (Fig 1C). To be used in sunlight, a white LED was mounted on the instrument; the distance between the white LED and CMOS sensor was 7 cm (Fig 1B). The light emitted by the LED through the cell sample was shadowed on the sensor array. Then, the images captured by the sensor were sent to a personal computer (PC) through a USB 2.0 interface and processed by image processing software (Matlab version 7.9.0). The image processing software contained three algorithms, including the automatic segmentation algorithm, the PPED feature vector extraction algorithm and the cell classification algorithm.

**Fig 2:**
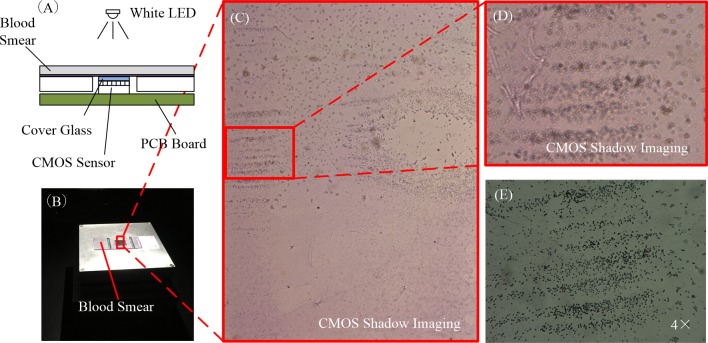
CMOS shadow imaging system. (A) Diagram of blood smear shadow image captured by this instrument. (B) Photo of blood smear shadow image captured by this instrument. (C) Raw image of the blood smear captured by the instrument. (D) Enlarged image of the red box area in Fig 2C. (E) The same area as Fig 2D captured by a 4× microscope objective.

The images of blood smear captured by shadow imaging system were unlike the images (Fig 2C and 2E) captured by a microscope. In the shadow image system is a lens-less system, and there was a short distance between the blood sample and the CMOS image sensor. As a result, diffraction when the visible light passed through the blood sample to the sensor blurred the image of the cells. The diffraction image (Fig 2D) of the blood smear captured by the CMOS image sensor, it clearly shows that the diffraction had a significant impact. The diffraction image of the cells obtained by the system was not clear like the image captured with microscopy; it was blurry. Fortunately, according to Fourier optics, a diffraction image contains the same information as a non-diffraction image, and this information could be recovered by the mathematical modeling of non-diffraction images. Different types of WBCs also had different diffraction patterns. There was diffraction in the images of the cells, but the distance from the sample to the sensor was only 200 μm. Even though the light source was incoherent, the instrument could still collect the general pattern of the cells.

The image of the blood smear captured by the CMOS system was similar to the image captured by the 4× objective lens in resolution; the magnification of our system was similar to that obtained with a 4× microscope objective. By comparing the figures (Fig 2D and 2E), there was a more obvious diffraction phenomenon in the image captured by the shadow image system. In addition, blood cells in the blood smear should be few and scattered. To eliminate the diffraction phenomena, we must move the sample as close to the surface of the image sensor as possible. However, there was a cover glass on the CMOS sensor to protect the sensor array and integrated circuit. The distance between the sample to the sensor surface, that is, the thickness of cover glass, was only 0.2 mm. The diffraction phenomenon was not obvious, and it was still able to classify WBCs.

### Preparation of blood smears

The study was approved by the School of Automation and Information Ethics Committee, Xi’an University of Technology. Participants had provided their verbal informed consent to participate in this study, and the procedure had approved by the School of Automation and Information Ethics Committee. The participants were the clinical patients of Xi’an University of Technology Hospital, and the clinicians had verbal informed the participants that their whole blood would be used for this study after routine blood tests, all participants had agreed. All blood samples had already been made routine blood tests for disease diagnoses, and the blood samples we used was the residual whole blood and would discard. In order to facilitate participants, the clinicians only obtained their verbal informed consents and recorded them on the patient records. For participants’ privacy, the blood samples and the test reports had hidden the participants’ name, age and other private information.

The specimens were treated with Wright’s stain [16], and different types of WBCs had different characteristics in the images. Patient blood samples had approximately 4 × 10^6^~5 × 10^6^ cells/mL, which was a very high concentration for the shadow imaging system. In our system, the shadow images were blurred by diffraction, and the images of the cells would be polluted if another cell were too close. Therefore, if the two cells were too close, the individual images (Fig 2D) would be mixed together. Due with this problem, the whole blood was diluted with PBS (Saline 1X, PO_4_ 0.0067M), 20 μL human whole blood was mixed with 60 μL PBS.

To identify the WBCs, the whole blood was stained using the Wright-Giemsa method [17]. A drop of blood was taken from the patient and smeared on a glass slide. To obtain a smear with proper thickness, the spreading slide was held at an angle greater than 45°. The blood smear was stained with Wright-Giemsa stain for 3 min, and then large amounts of distilled water were added to the stain. The solution was mixed by gentle blowing every few minutes, and then the stain was gently washed off under running water for 30 s. To dry, the slide was shaken off and air-dried for an additional 5 min. Finally, the blood smear was used for shadow image capture.

### Automatic segmentation algorithm

First, the images of the WBCs were segmented from the blood smear images by an automatic segmentation algorithm. The color obtained by the shadow imaging system was inaccurate, and RGB images were converted to grayscale images. Then, the watershed segmentation algorithm was used to segment the blood smear images. From each blood smear image, a few to dozens of WBCs could be collected. From these segmented images of WBCs, it was easy to measure the size and count the number of WBCs. However, because of the low resolution of the blood smear, a simple and effective algorithm should be used for image segmentation. Fortunately, the WBCs and RBCs in the blood smear looked different as grayscale images. The grayscale of the WBCs were usually lower than 95, and the algorithm could easily carry out segmentation based on this characteristic. The watershed algorithm [18] was used to mark connected domains, and then each WBC image was saved as a picture.

### Image recognition algorithm

In this paper, a new image representation algorithm, “Projected Principal-Edge Distribution” (PPED), was used [15]. The algorithm was developed for use in the image recognition system hardware based on the neural associative processors, and the computing speed can be greatly improved. The PPED algorithm was a type of feature recognition algorithm that is sensitive to edge shape, and the algorithm was simple to actualize.

The PPED features were extracted from the WBC images, which were segmented by the method mentioned above. The resolution of the images of WBCs captured by the CMOS shadow imaging system was low. The PPED algorithm was demonstrated to be a useful method to sort the three subtypes of WBCs in this system. To verify the algorithm, the 20×, 10×, and 4× objective lenses of the microscope were used as a reference system to compare the proposed shadow imaging instrument. Therefore, the PPED algorithm could be used to extract the image feature vector, and then it could be used for WBCs classification.

The images of the cells were segmented, and then the interpolation method was used to expand the resolution of these images to 64×64 pixels, which was called I(x, y). Then, I(x, y) carried out spatial filtering by taking the convolution of 5×5 pixel data I(x, y) and 5×5 kernel as

$$I_{d}^{*}=|\sum_{p=-2}^{2}\sum_{q=-2}^{2}K_{d}(p,q)\times I(x+p,y+q)|.$$ (1)

Here, a threshold value TH(x, y) was introduced to detect an edge. The edge flag F_d_(x, y) was obtained by comparing the maximum value of the I_d_^*^ and the threshold value TH(x, y) shown as

$$F_{d}(x,y)=\left\{ \begin{array}{l} 0:\;If\;\max_{d}\left\{I_{d}^{*}(x,y)\}<TH(x,y\right)\\ 1:\;If\;\max_{d}\left\{I_{d}^{*}(x,y)\right\}\geq TH(x,y) \end{array}\right..$$ (2)

The bit map of each edge flag was called the feature map. The horizontal edge flags and the vertical edge flags were obtained as

$$P_{H}(j)=\sum_{i=0}^{63}F_{H}(i,j),$$ (3)

$$P_{V}(i)=\sum_{j=0}^{63}F_{V}(i,j).$$ (4)

The + 45° and the—45° edge flags were obtained as

$$P_{\pm 45{}^{\circ}}(m)=\sum_{m=i+j}F_{\pm 45{}^{\circ}}(i,j),$$ (5)

where m was an integer from 0 to 126. In a similar manner, a ±45° oblique projection sum edge marker was obtained. Regarding the diagonal projection sums P_±45°_’s, reduction is carried out in two steps. In the first step, 127 P_d_’s are reduced to 64 diagonal projection sums by taking an average as:

$$P_{d}^{\prime }(m)=\frac{P_{d}(2m-1)}{2}+P_{d}(2m)+\frac{P_{d}(2m+1)}{2}\;d\in \{+45{}^{\circ},-45{}^{\circ}\},$$ (6)

where m runs from 0 to 63 and *P*_*d*_(−1) = *P*_*d*_(127) = 0. In the second step, the 64 diagonal projection sums (P_H_, P_+45°_, P_V_, and P_-45°_) thus, obtained are then reduced to 16 diagonal projection sums by just merging four neiboring sums to one as

$$P_{d}^{*}(j)=\sum_{r=0}^{3}P_{d}[4j+r]\;d\in \{H,V,+45{}^{\circ},-45{}^{\circ}\},$$ (7)

**Fig 3:**
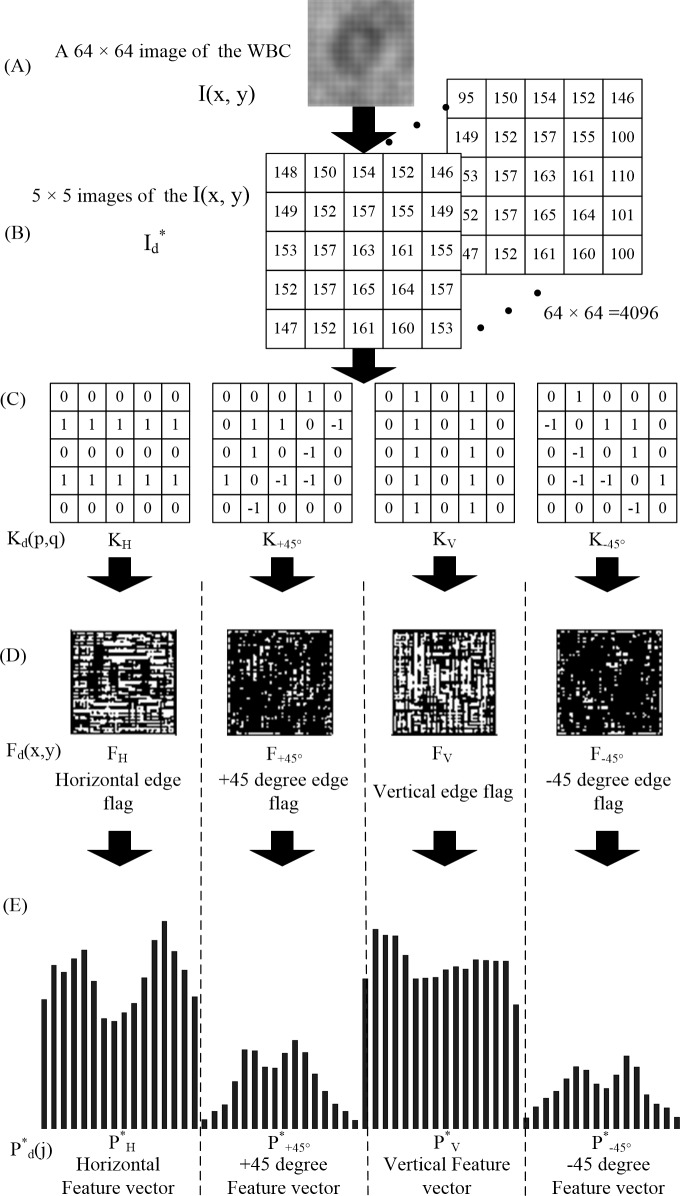
Generation of the PPED vector. (A) Image of WBC captured by CMOS shadow imaging instrument. (B) A five-by-five matrix of the WBC image, every pixel of the image would create a matrix. (C) Filtering kernels for detecting four principal edges. (D) Edge flag of four directions. (E) Sixty-four-dimensional feature vector of the WBCs.

The 64 diagonal projection and is reduced to 16 diagonal projections and merged with adjacent and four. The four sets of projection sums, P_H_, P_+45°_, P_V_, and P_-45°_, were the series connected in this order, and a 64-dimensional PPED vector was finally obtained (Fig 3).

The 64-dimension feature vector of the cell images was the final result of the PPED algorithm. However, the resolution of the images of WBCs obtained by the proposed system was low. Then, a novel algorithm was used to sort the three subtypes of WBCs.

### WBC classification algorithm

A method to classify WBCs compared the distance between two feature vectors of the cells. The smaller the distance between the two feature vectors, the more likely the two cells were the same type. It was calculated using the Euclidean distance between two vectors.

Euclidean distance:

d(x,y)=[∑i=1n(xi−yi)2]12 (8)

**Fig 4:**
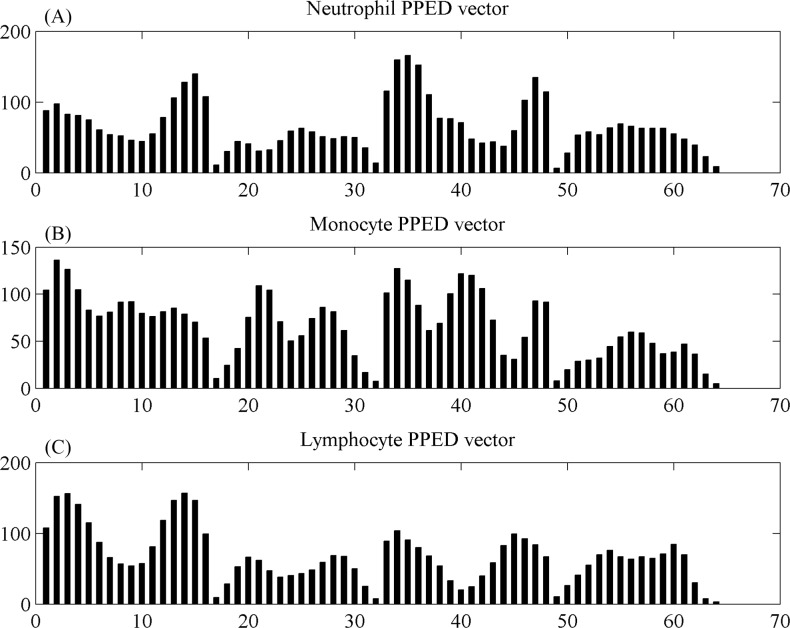
Standard PPED feature vectors of the three subtypes of WBC. (A) PPED vector of neutrophils. (B) PPED vector of monocytes. (C) PPED vector of lymphocytes.

In this paper, the classification method that measured the distance between the PPED feature vector of each cell and the three subtypes of standard PPED feature vectors (Fig 4) was used, and the standard PPED vector. The high-resolution images of the WBCs captured by the 10× objective lens were used to recognize three subtypes of leukocytes manually. The low-resolution images of WBCs captured by the 4× objective lens were used to extract the standard PPED vector. The low-resolution images of the WBCs could not be used to easily recognize the subtypes of WBCs manually, but the high-resolution images of the same cell could. Finally, the standard PPED vectors of the three subtypes were calculated by the PPED method mentioned in a previous paper, and the standard PPED feature.

The images of the WBCs captured by the CMOS shadow imaging system had low resolution, and other features should be added to improve the accuracy of the WBC classification. The size of the WBCs was a useful and important feature, which was close to the edge of the image and reduced the false recognition rate, providing a more accurate classification of the WBCs. The Euclidean distance of each cell to the standard vector was shown as

Dp(i)=[∑m=063(Pd(j)−Vm(j))2]12i∈{Neu,Mon,Lym}. (9)

Here, D_p_ (i) was the Euclidean distance of the ith cell PPED vector from the standard vector. The subscript m indicates the three subtypes of WBCs, neutrophil (Neu), monocyte (Mon) and lymphocyte (Lym). However, the low-resolution images lead to inaccurate classification of the WBCs. The missing details of the cell image, caused by low resolution, made the feature extraction of different types of cells difficult. Therefore, we propose a novel algorithm that normalizes the distance between PPED and the standard vector center and then converts the value to probability:

$$D_{m}(i)=\frac{D_{p}(i)}{argmax(D_{p}(i))}\;i\in \{Neu,Mon,Lym\},$$ (10)

$$P_{m}(i)=1-D_{m}(i)\;i\in \{Neu,Mon,Lym\}.$$ (11)

Here, D_m_(i) was the normalized Euclidean distance and the P_m_(i) was the probability of the three subtypes of WBCs. The largest, the median and the smallest sized WBCs were the neutrophil, monocyte and lymphocyte, respectively. By the size of the cell, it was preliminary to determine which types the cell belongs to, the method shown as

$$t=\left\{ \begin{array}{c} Lym,\;5\mu m<d<10\mu m\\ Neu,10\mu m<d<14\mu m\\ Mon,14\mu m<d<25\mu m \end{array}\right..$$ (12)

Here, t was the types of the WBCs and d was the diameter of the WBCs. Then, the probability Ps(i) distinguished by the size of WBCs was shown as

$$P_{s}(i)=\left\{ \begin{array}{c} 1,\;i=t\\ 0,\;i\neq t \end{array}\right.\;i,t\in \{Neu,Mon,Lym\}.$$ (13)

This probability P_m_(i) accounts for 90% of the weight of the judgment, and the probability P_s_(i) accounts for 10%. The function of different types of WBC classifications was shown as

$$T_{m}(i)=0.9\times P_{m}(i)+0.1\times P_{s}(i)\;i\in \{Neu,Mon,Lym\},$$ (14)

where T_m_(i) was the probability of the three subtypes of WBCs. For every sample, three T_m_(i) values could be calculated, which show neutrophils, monocytes and lymphocytes. Which T_m_(i) value was the largest, which corresponded to the type of cells.

## Results and discussion

**Fig 5:**
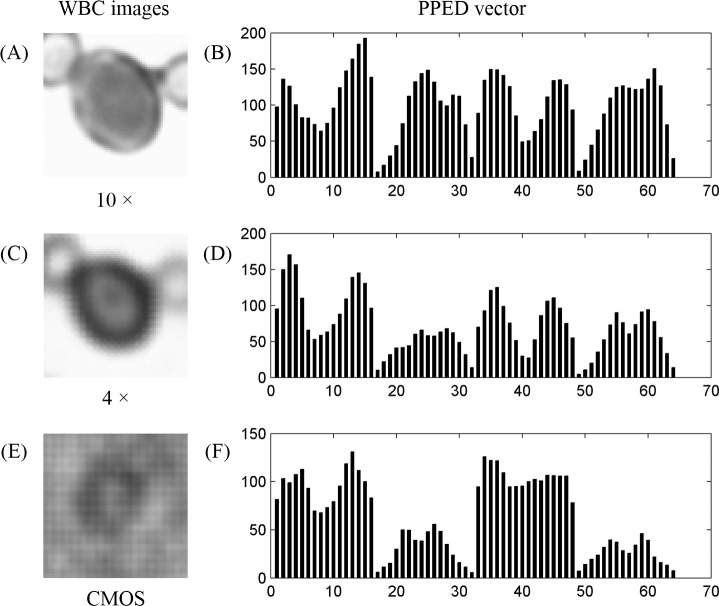
Images of WBCS and the PPED vector at different resolutions. (A) Cell image with a 10× objective lens. (B) PPED vector of Fig 5A. (C) Same cell image with 4× objective lens. (D) PPED vector of Fig 5C. (E) Same cell image by CMOS shadow imaging instrument. (F) PPED vector of Fig 5E.

The feature vector of the image of a monocyte obtained with a 10× objective lens and the amplification effect of the shadow image of the lens-free shadow imaging system were similar to those obtained with a 4× objective lens. However, the low amplification of the image indicates low resolution, and the lower the resolution of the image, the less information provided. Fortunately, experiments demonstrated that the PPED was still useful with the low-resolution image. We used the 10× and the 4× objective lenses to capture two images of the same cell and extract the PPED vector (Fig 5B and 5D).

Here, the images captured by the 10× and 4× objective lenses were only different in resolution, so we called the image captured by the 10× objective lens high resolution and the image captured by the 4× objective lens low resolution. Although the PPED vectors of the high- and low-resolution images were not exactly the same, they were similar.

Another problem was that the shadow image of the blood cells was disturbed by diffraction in the CMOS shadow imaging system. Therefore, an experiment was conducted to demonstrate that the diffractive images contained all information. The PPED vectors of the shadow images of WBCs were similar to the images obtained by microscopy. The instrument brought the cell sample close to the surface of the CMOS image sensor so that the diffraction phenomenon was small, and the shape information of the cell was preserved. Moreover, the treatment of cell samples was relatively simple; the blood cells needed to be stained, but the RBCs did not need to be dissolved. Three characteristic vectors (Fig 5E and 5F) of the same cell with different resolutions were extracted.

The images captured by the 10× and 4× objective lenses were only different in resolution, and the images captured by the 4× objective lens and the CMOS shadow imaging system were similar in resolution. The PPED vectors (Fig 5D and 5F) were similar. Therefore, the performance of the proposed system was comparable to that of the 4× objective lens microscope in the classification of WBCs.

To verify the performance and reliability of the proposed instrument and algorithms, an experiment was conducted. The whole blood specimens collected from six outpatients were tested by the CMOS system and the reference system, a hematology analyzer (BC-5180, Mindray, Shenzhen, Guangdong, China). The result from the CMOS system and the reference system had a relatively good correlation, shown in Table 1.

**Table 1:** Classification results of WBCs from patient samples.

Sample no.	Method	Neutrophil (%)	Monocyte (%)	Lymphocyte (%)	Correlation
**1**	CMOS	61.2	21.8	17.7	0.97
	BC-5180	68.5	11.2	20.3	
**2**	CMOS	55.2	15.1	29.7	0.89
	BC-5180	52.6	4.9	42.5	
**3**	CMOS	67.4	5.7	27	1
	BC-5180	68	5.7	26.3	
**4**	CMOS	56	6.67	37.33	1
	BC-5180	55.2	9	35.8	
**5**	CMOS	66.35	11.85	21.8	0.927
	BC-5180	60.8	4.8	34.4	
**6**	CMOS	62.77	11.7	25.53	0.95
	BC-5180	58.9	5.6	35.5	

**Fig 6:**
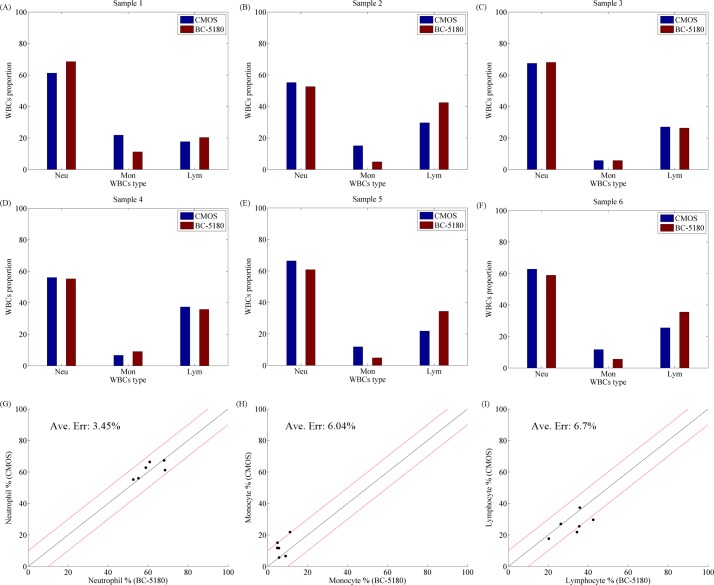
Results of characterization of three whole blood specimens. (A) Comparison of the BC-5180 (Mindray, Shenzhen, Guangdong, China) and CMOS systems for the WBC population in sample 1. (B) Comparison of the BC-5180 (Mindray) and CMOS systems for the WBC population in sample 2. (C) Comparison of the BC-5180 (Mindray) and CMOS systems for the WBC population in sample 3. (D) Comparison of the BC-5180 (Mindray) and CMOS systems for the WBC population in sample 4. (E) Comparison of the BC-5180 (Mindray) and CMOS systems for the WBC population in sample 5. (F) Comparison of the BC-5180 (Mindray) and CMOS systems for the WBC population in sample 6. (G) Comparison of the CMOS and BC-5180 (Mindray) systems for the neutrophil population in six samples. (H) Comparison of the CMOS and BC-5180 (Mindray) systems for the monocyte population in six samples. (I) Comparison of the CMOS and BC-5180 (Mindray) systems for the lymphocyte population in six samples.

Because the correlation between the two types of expression forms was not intuitive, the data in the table are shown as a histogram in Fig 6. It is clear that the classification results of both the CMOS and reference systems had a strong correlation.

Here, the results of the classification the WBCs in the whole blood samples, which were collected from six patients by the hospital, are clear. Furthermore, the linear fit (Fig 6G–6I) of each type of WBCs is shown by Matlab. To evaluate the efficiency of the proposed instrument, the accuracy for each type of WBCs was also calculated. Here (Fig 6G), the comparison between the proposed system (CMOS) and the reference system (BC-5180, Mindray) for the percentage of neutrophils had a mean error of 3.45%. The same analyses were also performed for the other types of WBCs (Fig 6H and 6I) for monocytes and lymphocytes, and the mean errors were 6.04% and 6. 7%.

## Conclusion

In summary, a WBC classification method based on shadow imaging of a CMOS sensor was proposed. The system contained inexpensive parts, such as the CMOS sensor, a white LED and an aluminum alloy shell, and the total cost of this system was below $100. A white LED was used in the proposed system, which means that the LED could be replaced by sunlight. The system power consumption would be significantly reduced and more conducive to POCT with sunlight. Due to the algorithms proposed in this paper, the system was not only cheaper but also automated. For the blood tests from whole blood samples from six outpatients (proportion of WBCs), the result obtained was a mean correlation index of 0.96. The mean errors of percentage of neutrophils (3.45%), monocytes (6.04%) and lymphocytes (6.7%) were lower than the method proposed by Mohendra Roy [14]. Although the performance was still less than traditional methods, it was sufficient to demonstrate that the proposed instrument and classification method were effective. The instrument was 10 × 10 × 10 cm in volume, but the aluminum alloy shell could be further reduced to chip size. The widespread application of the instruments would provide a more convenient method of routine blood examination.
